# Comparison of free and bound phenolic compositions and antioxidant activities of leaves from different mulberry varieties

**DOI:** 10.1186/s13065-021-00747-0

**Published:** 2021-03-29

**Authors:** Zhenjiang Wang, Cuiming Tang, Gengsheng Xiao, Fanwei Dai, Sen Lin, Zhiyi Li, Guoqing Luo

**Affiliations:** 1 Sericulture & Agri-Food Research Institute, Guangdong Academy of Agricultural Sciences, Guangzhou, China; 2 Key Laboratory of Urban Agriculture in South China, Ministry of Agriculture and Rural Affairs, Guangzhou, China

**Keywords:** Bound Phenolics, Antioxidant properties, Mulberry leaf, Free Phenolics, Mulberry variety

## Abstract

Mulberry leaves are used in traditional Chinese medicine and contain numerous active substances that are known to be beneficial for human health. The aim of this study was to investigate the phenolic compositions and antioxidant activities of the leaves from 23 mulberry cultivars. Qualitative LC-ESI-QTOF analysis revealed the presence of 11 phenolic compounds in the free phenolic extracts and 10 phenolic compounds in the bound fractions. Chlorogenic acid and caffeic acid were the major components in the free and bound fractions, respectively. The results revealed that the changguosang cultivar from Taiwan contained the greatest content of phenolic compounds as well as the highest antioxidant activity among the 23 cultivars examined, as determined using three separate antioxidant assays. The isoquercitrin, chlorogenic acid, and rutin contents of the free phenolic extracts displayed significant correlations with the antioxidant activities, while syringic acid and rutin were the main contributors to the antioxidant activities of the bound phenolic fractions. The obtained results demonstrate that mulberry leaves contain a variety of beneficial phenolic substances and may be suitable for further development as a herbal medicine.

## Introduction

Phytonutrients play important roles in improving human health and may protect against heart disease, cancer, the effects of aging, and membrane damage. In particular, phytonutrients possessing antioxidant properties can inhibit the propagation of free-radical reactions implicated in the development of aging-related diseases. Consequently, numerous studies have been conducted to elucidate the characteristics and roles of antioxidant compounds from plants. Antioxidants can delay or suppress the oxidation of molecules by inhibiting the activation or propagation of oxidative chain reactions or scavenging the free radicals generated during oxidative processes [37]. The antioxidant activity of plant tissues is primarily attributable to phenolic, etc. and a high correlation was observed between the antioxidant activity and the total phenolic content of the extract. [1]. Hence, phenolic compounds have attracted considerable attention as potential protective factors against cancer and heart disease owing to their antioxidant activities [8].

Mulberry (*Morus alba* L.) is a moraceous plant that is extensively cultivated throughout Asia to feed silkworms during the commercial production of silk. It is distributed throughout temperate to subtropical and tropical regions and can be grown under a wide range of climatic, topographic, and soil conditions [11]. Mulberry leaves are considered a nutritious, palatable, and safe food or food additive containing carbohydrates, proteins, calcium, iron, β-carotene, and vitamin B1 [6] and are also a rich source of phenolic compounds such as caffeic acid, rutin, quercetin, isoquercitrin, and astragalin [9, 23]. Mulberry leaves are commonly used as antidiabetic, hypolipidemic, antihypertensive, anti-atherosclerotic, and anticonvulsant agents [4]. These pharmacological effects of mulberry leaves are closely related to their phenolic composition [14]. Plant phenolic compounds have been reported to play a preventive role against various diseases owing to their remarkable antioxidant, antimicrobial, and other activities [7, 29]. For example, Wu et al. [38] reported that mulberry leaf phenolic extract decreases hepatic lipid accumulation via activation of the AMP-activated protein kinase signaling pathway.

There is strong evidence for the variation of phenolic content and antioxidant activity among different species of plants belonging to the same genus [30, 35]. Thus, the identification and quantification of phenolic compounds can provide crucial information regarding the antioxidant function, food quality, and potential health benefits of a specific plant. G1, G6, G8, and GSW are from North China; B-2–8, R2, 7403, and DS are from South China; Z1, Z2, and Z4 are from Southwest China; CGS is from Taiwan; J4-1 and J5 are from Japan; T6, T7, BR60, S54, and QM are from Thailand; Y2 and YXM2 are from India; and Y1 and YD are from Vietnam. Therefore, the aim of this study was to determine and compare the phenolic compositions and antioxidant activities of these 23 samples of mulberry leaves.

## Materials and methods

### Mulberry leaf samples and preparation

All of the mulberry varieties examined in this study were cultivated in an experimental field in Guangzhou, which was managed by the South China branch of the National Mulberry Germplasm Resource Garden. Mulberry leaves are generally picked in the spring and late autumn frost period, and it is better to pick them in the morning and evening. Leaves from each mulberry variety were harvested, washed with distilled water, and air-dried at 55 °C in a thermostatic hot air drying oven for 7–9 h. The dried leaves were ground into powders using a high-speed pulverizer and stored in airtight containers at − 20 °C prior to analysis [35]. The different mulberry varieties examined are listed in Table 1.

**Table 1:** Names, species, and abbreviations of the mulberry varieties examined in this study

Cultivar	Species	Abbreviation
Gu 1	*M. alba* L	G1
Gu 6	*M. alba* L	G6
Gu 8	*M. alba* L	G8
Gusangwang	*M. mongolica* var. *diabolica* Koidz	GSW
Bei-2-8	*M. atropurpurea* Roxb	B-2-8
R2	*M. atropurpurea* Roxb	R2
7403	*M. atropurpurea* Roxb	7403
Dashi	*M. atropurpurea* Roxb	DS
Zangjiangxin 1	*M. mongolica* C.K.Schneid	Z1
Zangjiangxin 2	*M. mongolica* C.K.Schneid	Z2
Zangjiangxin 4	*M. mongolica* C.K.Schneid	Z4
Changguosang	*M. rotundiloba* Koidz	CGS
JP4-1	*M. multicaulis* Koidz	J4-1
JP5	*M. multicaulis* Koidz	J5
TL6	*M. rotundiloba* Koidz	T6
TL7	*M. rotundiloba* Koidz	T7
BR60	*M. rotundiloba* Koidz	BR60
S54	*M. rotundiloba* Koidz	S54
Qingmai	*M. rotundiloba* Koidz	QM
Yin 2	*M. serrata* Rox	Y2
Yinximeng 2	*M. serrata* Rox	YXM2
Yue 1	*M. rotundiloba* Koidz	Y1
Yueda	*M. rotundiloba* Koidz	YD

### Chemicals and reagents

Acetone, *n*-hexane, ethyl acetate, concentrated sulfuric acid, sodium hydroxide, phosphoric acid, acetonitrile, ascorbic acid, methanol, formic acid, sodium acetate, acetic acid, hydrochloric acid, iron(III) chloride hexahydrate solution, ammonium acetate, were purchased from Guangzhou Chemical Reagent Factory (Guangzhou, China). phenolic compound standards [rutin (Rut), quercetin (Que), quercitrin (Quer), isoquercitrin (Iso), chlorogenic acid (ChA), syringic acid (SyA), ferulic acid (FeA), caffeic acid (CaA), resveratrol (Res), epicatechin (Epi), astragalin (Ast), scopoletin (Sco), galangal (Gal), catechuic acid (CatA), vanillic acid (VaA), benzoic acid (BeA), gallic acid (GaA), and protocatechuic acid (PrA)] were purchased from the National Institutes for Food and Drug Control (Beijing, China). 2,4,6-tris(2-pyridyl)-*S*-triazine (TPTZ), 1,1-diphenyl-2-picrylhydrazyl (DPPH), 2,2ʹ-Azino-bis(3-ethylbenzothiazoline-6-sulfonic acid) diammonium salt (ABTS), and 6-hydroxy-2,5,7,8-tetramethylchroman-2-carboxylic acid (trolox) were purchased from Sigma-Aldrich (St. Louis, MO, USA).

### Extraction of free phenolic compounds

It was necessary to remove lipids from the mulberry leaves prior to extraction of the free phenolic compounds [12]. Considering that the lipid content of mulberry leaves is not high, the degreasing procedure in this study was adjusted. Free phenolic compounds were extracted according to a modified version of the methods reported by Li et al. [20] and Kim et al. [16]. Briefly, samples of the dried leaves (10 g) were extracted with *n*-hexane (250 mL) for 10 min under continuous stirring (10,000 rpm) in an ice bath, followed by centrifugation (5000 rpm, 5 min) and removal of the supernatant. The residue was added to chilled acetone/water (8:2, v/v, 250 mL) followed by homogenization (5000 rpm, 5 min) and centrifugation (5000 rpm, 5 min). The residue was extracted again under the same conditions. The two supernatants were combined and evaporated to dryness at 50 °C on a rotary evaporator (RE-52AA, Shanghai Yarong Biochemical instrument Factory, Shanghai, China). The dried samples were dissolved in methanol/water (8:2, v/v), filtered through 0.22 μm membrane filters, and stored at − 80 °C prior to analysis. Each sample was extracted in duplicate. The residue was used to measure the bound phenolic compounds, as described in the following subsection.

### Extraction of bound phenolic compounds

The bound phenolic compounds were extracted by alkaline hydrolysis according to previously described procedures [5, 18, 21] with some modifications. Briefly, the mulberry leaf residues obtained after extraction of the free phenolic compounds were hydrolyzed in 2 M NaOH (100 mL) at room temperature for 1.5 h with continuous stirring under nitrogen atmosphere. The resulting mixtures were acidified to pH 2 with 6 M HCl and then extracted six times with ethyl acetate (120 mL each time). The ethyl acetate fractions were combined and evaporated to dryness at 45 °C on a rotary evaporator (RE-52AA). The dried extracts were redissolved in 80% methanol, filtered through 0.22 μm membrane filters, and stored at − 80 °C prior to analysis.

### LC-ESI-QTOF analysis

Samples were analyzed by LC-ESI-QTOF according to a modified version of the method described by Tomas et al. [36]. Each sample was vortexed for 30 s, filtered through a 0.22 μm organic membrane, and transferred into an injection vial. The temperature of the chromatographic column was set to 35 °C, and the injection volume was 1 μL. The mobile phase consisted of 0.1% formic acid in water (solvent A) and 0.1% formic acid in acetonitrile (solvent B) in positive ion mode or 2 mM ammonium acetate in water (solvent A) and acetonitrile (solvent B) in negative ion mode. The flow rate was set to 400 μL/min with a gradient from 5 to 95% solvent B over 22 min, as shown in Table 2. The Agilent 6545A QTOF mass spectrometer is controlled by the control software (LC/MS Data Acquisition, Version B.08.00) based on the Auto MS/MS mode for primary and secondary mass spectrometry data acquisition. The MS and MS2 acquisition rates were 5 and 10 spectra per second, respectively. The secondary collision energy is 0v and 10v respectively.Eight ions were selected in the first-level spectrum for the second-level scan. The m/z range of primary and secondary mass scanning are both 50–1100. Spectra were collected in both positive and negative modes, and the acquired data were saved in centroid format. The ESI ion source parameters were as follows: ion source gas temperature, 320 °C; nitrogen flow rate, 8 L/min; sheath gas flow rate, 12 L/min; sheath gas temperature, 350 °C; capillary voltage, 4000 V (positive ion mode) or 3500 V (negative ion mode).

**Table 2:** LC-ESI-QTOF mobile phase gradient

Time (min)	Flow rate (μL/min)	A (%)	B (%)
0	400	95	5
1.5	400	95	5
2.5	400	90	10
14	400	60	40
22	400	5	95
25	400	5	95
26	400	95	5
30	400	95	5

### Determination of phenolic content using HPLC

The phenolic compounds present in the 23 mulberry leaf samples were determined by HPLC analysis (Agilent 1200, Agilent Technologies Inc., Karlsruhe, Germany) according to a modified version of the methods reported by Eom et al*.* [10] and Subhashinee et al. [33]. Chromatographic separation was performed using an Capcell Pak ADME column (250 × 4.6 mm, 5 μm). The mobile phase consisted of phosphoric acid/water (0.2:100, v/v, solvent A) and acetonitrile (solvent B). The solvent gradient was as follows: 0–20 min 10% B, 20–30 min 16% B, 30–40 min 16% B, 40–60 min 20% B, 60–70 min 30% B, 70–71 min 35% B, 71–75 min 80% B, 75–76 min 80% B, 76–90 min 10% B. The solvent flow rate was 1.0 mL/min, the column temperature was set to 25 °C, and chromatograms were recorded at 280 nm and 350 nm.

Dionex Acclaim 120 C-18 analytical column.

CAPCELL PAK ADM.

### Determination of FRAP activity

The ferric reducing antioxidant power (FRAP) assay was performed according to a modified version of a previously reported method [39, 40]. A working solution was prepared by mixing 10 mL of 300 mM acetate buffer (0.1870 g of sodium acetate and 1.6 mL of acetic acid), 1 mL of TPTZ solution (10 mM TPTZ in 40 mM HCl), and 1 mL of 20 mM iron(III) chloride hexahydrate solution. This mixture was pre-warmed to 37 °C and should always be prepared fresh. Samples of the mulberry leaf extracts (100 μL) were incubated with 3.0 mL of the FRAP reagent for 4 min at 25 °C. The absorbance at 593 nm was then measured using a spectrophotometer (UV-1700, Shimadzu Instruments Manufacturing, CO., LTD, Suzhou, China). The FRAP values were expressed as μmol Fe^2+^ equivalents per gram of dry weight (μmol Fe^2+^/g DW).

### Determination of DPPH radical scavenging activity

The DPPH radical scavenging activities of the mulberry leaf samples were determined as described in previous reports [2, 3, 22]. Briefly, the mulberry leaf extracts and DPPH solutions were diluted to appropriate concentrations, and a solution of ascorbic acid in methanol was used to prepare a standard curve (*R*^2^ = 0.993). Next, 1 mL of the diluted sample was mixed with 5 mL of 2 mM DPPH solution in methanol. The mixture was stirred vigorously and incubated for 50 min in the dark at room temperature. The absorbance at 520 nm was then measured using a UV2300II spectrophotometer and the results were expressed in μmol of ascorbic acid equivalent antioxidant capacity (AEAC) per gram of dry weight (μmol AEAC/g DW).

### Determination of ABTS radical scavenging activity

The ABTS radical scavenging activities of the mulberry leaf samples were determined as described in previous reports [27, 31, 34]. Briefly, an ABTS stock solution was prepared by mixing 7 mM ABTS and 2.45 mM potassium persulfate (1:1, v/v) followed by incubation for 16 h in the dark at room temperature; the resulting ABTS radical solution was used within 24 h. The ABTS stock solution was diluted with methanol until its absorbance at 734 nm reached 0.70 ± 0.02. Next, 100 μL of the diluted sample was mixed with 3.8 mL of the ABTS working solution and the absorbance at 734 nm was measured after incubation at room temperature for exactly 6 min. Trolox was used as a reference to generate a standard curve (*R*^2^ = 0.995), and the results were expressed as μmol of trolox equivalent antioxidant capacity (TEAC) per gram of dry weight (μmol TEAC/g DW).

## Results and discussion

### Qualitative analysis of phenolic compounds in mulberry leaves

To determine the presence of various biologically active phenolic compounds in the mulberry leaf samples, the obtained mass spectra of the samples were compared with spectra of standard compounds with respect to retention time (RT), molecular ion peak, and structural fragments observed in the secondary mass spectra.

In LC–MS, a sample is first subjected to liquid chromatography to separate the sample components, which are subsequently ionized and separated according to their mass-to-charge ratios to reveal information regarding the molecular weight, structure, and component amount.

Mulberry leaves are rich in a variety of phenolic compounds, the composition and content of which can vary for mulberry leaves from different cultivars and regions. LC-ESI-QTOF analysis revealed the presence of 11 highly matched phenolic substances in the free phenolic extracts and 10 phenolic substances in the bound phenolic extracts, as summarized in Tables 3 and 4, respectively. The free phenolic compounds detected in the mulberry leaf samples were BeA, PrA, GaA, CaA, Sco, Epi, Que, ChA, Iso, Rut, and Ast, while the bound phenolic compounds were SyA, GaA, BeA, PrA, CaA, Iso, Rut, FeA, Ast, and Que. Among these compounds, Sco, Epi, and ChA were only detected in the free phenolic extracts, which indicates that these substances may be either absent from the bound phenolic extracts. In contrast, SyA and FeA were only detected in the bound phenolic extracts and not in the free phenolic extracts. Zou et al. [40] reported certain differences in the phenolic contents of different varieties of mulberry leaves, which is useful for helping farmers to select which varieties to cultivate for higher quality mulberry leaves. Qadir et al. [26] applied gas chromatography–mass spectrometry to identify and quantify the main phenolic compounds present in *M. alba* leaf extracts, including Que, GaA, and SyA. In the bound phenolic extracts, ChA was not detected, indicating that ChA may only exist in the free phenolic extracts. Most of the phenolic substances present in high contents in the free and bound phenolic extracts could be qualitatively analyzed by LC–MS.

**Table 3:** Qualitative analysis of free phenolic compounds in mulberry leaves

Compound	Molecular formula	Expected *m*/*z*	Observed *m*/*z*	Mass error (ppm)	RT (min)	Fragment ions (relative intensity, %)
BeA	C_7_H_6_O_2_	121.0295	121.0294	− 0.83	7.3186	121.02985 (100%), 77.04045 (14.2%), 82.49994 (1.9%)
PrA	C_7_H_6_O_4_	153.0193	153.0194	0.65	7.9276	153.01923 (100%), 153.04488 (3.3%), 109.02855 (2.6%)
GaA	C_7_H_6_O_5_	169.0142	169.0145	1.77	3.4645	169.01408 (100%), 125.02387 (3.3%), 61.7975 (2.5%)
CaA	C_9_H_8_O_4_	179.0350	179.0351	0.56	7.6609	179.03479 (100%), 135.04578 (5.3%)
Sco	C_10_H_8_O_4_	191.0350	191.0350	0	9.9880	191.03455 (100%), 176.01178 (9.3%), 147.02908 (3.5%)
Epi	C_15_H_14_O_6_	289.0718	289.0678	− 13.84	2.2628	243.0616 (100%), 289.06708 (41.6%), 244.06404 (12.5%)
Que	C_15_H_10_O_7_	301.0354	301.0356	0.66	13.8163	301.03537 (100%), 178.99699 (6.5%), 151.00305 (4.4%), 63.02433 (2.1%)
ChA	C_16_H_18_O_9_	353.0878	353.0865	− 3.8	6.7420	191.05605 (100%), 192.05885 (8.7%)
Iso	C_21_H_20_O_12_	463.0882	463.0903	4.53	8.6035	463.08832 (100%), 301.03555 (3%)
Rut	C_27_H_30_O_16_	609.1461	609.1478	2.79	8.9101	609.14642 (100%), 609.18945 (9%), 300.02475 (2.2%)
Ast	C_21_H_20_O_11_	447.0933	447.0942	− 2.01	10.6400	447.0932 (100%), 285.0388 (18.98%), 449.0994 (8.2%)

All spectra were recorded in negative ion mode with a collision energy of 10 V

**Table 4:** Qualitative analysis of bound phenolic compounds in mulberry leaves

Compound	Molecular formula	Expected *m*/*z*	Observed *m*/*z*	Mass error (ppm)	RT (min)	Fragment ions (relative intensity, %)
SyA	C_9_H_10_O_5_	197.0455	197.0457	1.01	3.3027	135.04504 (100%), 153.05786 (31.6%), 162.83853 (26.5%), 61.98833 (25.3%)
GaA	C_7_H_6_O_5_	169.0142	169.0144	1.18	3.4643	169.01404 (100%), 168.88683 (15.4%), 125.02482 (8.8%), 122.89381 (2.7%)
BeA	C_7_H_6_O_2_	121.0295	121.0305	8.26	7.3001	121.02959 (100%), 77.04009 (10.4%), 77.05341 (0.8%)
PrA	C_7_H_6_O_4_	153.0193	153.0193	0	7.7071	153.01918 (100%), 109.02876 (6.1%)
CaA	C_9_H_8_O_4_	179.0350	179.0352	1.12	7.7366	179.03502 (100%), 135.04532 (12.9%)
Iso	C_21_H_20_O_12_	463.0882	463.0877	− 1.08	8.5514	463.08813 (100%), 301.03839 (3.6%), 193.01373 (1.9%)
Rut	C_27_H_30_O_16_	609.1461	609.1469	1.31	9.2957	609.14612 (100%), 300.02649 (3.7%)
FeA	C_10_H_10_O_4_	193.0506	193.0508	1.04	10.0027	193.05028 (100%), 134.03656 (4.7%)
Ast	C_21_H_20_O_11_	447.0933	447.0937	0.89	10.9455	447.0921 (100%), 285.03876 (3.7%)
Que	C_15_H_10_O_7_	301.0354	301.0353	− 0.33	13.7901	301.03534 (100%), 300.99191 (5.2%)

All spectra were recorded in negative ion mode with a collision energy of 10 V

### Total phenolic contents

**Fig. 1:**
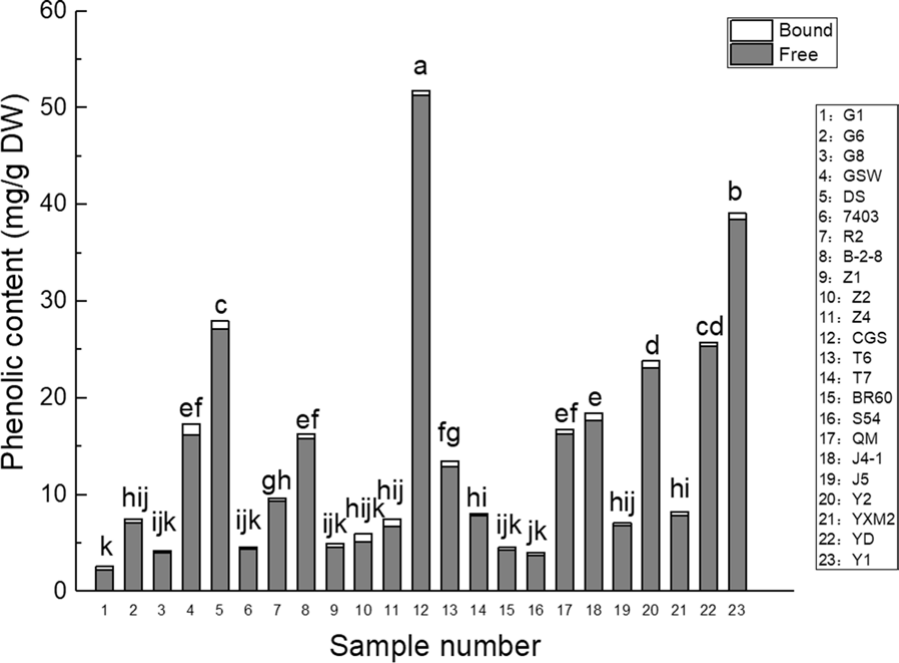
Free and bound phenolic contents of the 23 mulberry leaf samples

The total phenolic contents in the 80% acetone extracts of the 23 samples were measured by HPLC as presented in Table 5 and Fig. 1, revealing clear differences between the various cultivars. The total phenolic contents (including both free and bound compounds) increased in the following order: G1 (2.61 mg/g DW) < S54 (4.0 mg/g DW) < G8 (4.22 mg/g DW) < BR60 (4.55 mg/g DW) < 7403 (4.62 mg/g DW) < Z1 (4.97 mg/g DW) < Z2 (5.98 mg/g DW) < J5 (7.08 mg/g DW) < G6 (7.47 mg/g DW) < Z4 (7.50 mg/g DW) < T7 (8.07 mg/g DW) < YXM2 (8.24 mg/g DW) < R2 (9.66 mg/g DW) < T6 (13.49 mg/g DW) < B-2–8 (16.27 mg/g DW) < QM (16.75 mg/g DW) < GSW (17.3 mg/g DW) < J4-1 (18.46 mg/g DW) < Y2 (23.83 mg/g DW) < YD (25.72 mg/g DW) < DS (27.99 mg/g DW) < Y1 (39.12 mg/g DW) < CGS (51.81 mg/g DW).

**Table 5:** Total, free, and bound phenolic contents of the 23 mulberry leaf samples

Cultivar	Total phenolic content (μg/g DW)	Free phenolic content (μg/g DW)	Bound phenolic content (μg/g DW)
G1	2607.7 ± 161.35^ k^	2264.56 ± 161.35^ k^ (86.84%)	343.14 ± 46.25^ghi^ (13.16%)
G6	7472.27 ± 572.98^hij^	7147.13 ± 572.98^ijk^ (95.65%)	325.14 ± 43.63^ghi^ (4.35%)
G8	4224.72 ± 418.4^ijk^	4023.23 ± 418.4^ijk^ (95.23%)	201.49 ± 33.66^j^ (4.77%)
GSW	17,302.22 ± 1667.62^ef^	16,191.26 ± 1667.62^ef^ (93.58%)	1110.96 ± 105.93^a^ (6.42%)
DS	27,990.69 ± 2711.73^c^	27,156.6 ± 2711.73^c^ (97.02%)	834.08 ± 80.98b^c^ (2.98%)
7403	4621.51 ± 367.57^ijk^	4347.98 ± 367.57^ijk^ (94.08%)	273.53 ± 40.51^hij^ (5.92%)
R2	9660.23 ± 831.38^gh^	9322.52 ± 831.38^gh^ (96.5%)	337.71 ± 40.26^ghi^ (3.5%)
B-2–8	16,272.83 ± 1808.31^ef^	15,757.86 ± 1808.31^ef^ (96.84%)	514.97 ± 66.57^f^ (3.16%)
Z1	4965.79 ± 548.09^ijk^	4526.59 ± 548.09^ijk^ (91.16%)	439.19 ± 56.41^ fg^ (8.84%)
Z2	5984.99 ± 571.56h^ijk^	5110.54 ± 571.56^ijk^ (85.39%)	874.44 ± 95.62^b^ (14.61%)
Z4	7498.51 ± 682.07^hij^	6746.59 ± 682.07^ijk^ (89.97%)	751.92 ± 87.75^ cd^ (10.03%)
CGS	51,811.86 ± 5233.05^a^	51,279.3 ± 5233.05^a^ (98.97%)	532.56 ± 46.46^ef^ (1.03%)
T6	13,490.12 ± 1792.09^ fg^	12,954.33 ± 1792.09^ fg^ (96.03%)	535.78 ± 65.37^ef^ (3.97%)
T7	8068.84 ± 973.34^hi^	7828.8 ± 973.34^hi^ (97.03%)	240.04 ± 27.12^ij^ (2.97%)
BR60	4545.99 ± 456.22^ijk^	4300.87 ± 456.22^ijk^ (94.61%)	245.12 ± 27.41^ij^ (5.39%)
S54	4000 ± 397.67^jk^	3693.5 ± 397.67^jk^ (92.34%)	306.5 ± 34.73^hij^ (7.66%)
QM	16,748.36 ± 2401.89^ef^	16,240.99 ± 2401.89^ef^ (96.97%)	507.38 ± 53.43^f^ (3.03%)
J4-1	18,463.88 ± 2285.15^e^	17,712.43 ± 2285.15^e^ (95.93%)	751.45 ± 80.13^ cd^ (4.07%)
J5	7082.78 ± 636.06^hij^	6796.07 ± 636.06^ijk^ (95.95%)	286.71 ± 27.65^hij^ (4.05%)
Y2	23,833.07 ± 2884.06^d^	23,127.05 ± 2884.06^d^ (97.04%)	706.03 ± 74.29^d^ (2.96%)
YXM2	8235.79 ± 611.32^hi^	7849.57 ± 611.32^hi^ (95.31%)	386.21 ± 36.6^gh^ (4.69%)
YD	25,720.37 ± 3059.38^ cd^	25,347.54 ± 3059.38^ cd^ (98.55%)	372.83 ± 33.87^gh^ (1.45%)
Y1	39,120.86 ± 3514.82^b^	38,478.25 ± 3514.82^b^ (98.36%)	642.61 ± 60.36^de^ (1.64%)

Different letters indicate significant differences in total, free, and bound phenolic contents in 23 mulberry leaf samples (P < 0.05)

Values with no letters in common in each column are significantly different (P < 0.05), *n* = 3

Values in parentheses indicate the percentage contributions of the free and bound fractions to the total phenolic content for each sample

Table 5 also lists the free and bound phenolic contents and their corresponding percentages for the 23 samples. The contribution of the free phenolic fraction to the total phenolic content ranged from 85.39% (Z2) to 98.97% (CGS). The free phenolic content in the 23 varieties of mulberry leaves varied from 2.26 mg/g DW (G1) to 51.28 mg/g DW (CGS) and followed almost the same order as the total phenolic content: G1 (2.26 mg/g DW) < S54 (3.69 mg/g DW) < G8 (4.02 mg/g DW) < BR60 (4.3 mg/g DW) < 7403 (4.35 mg/g DW) < Z1 (4.53 mg/g DW) < Z2 (5.11 mg/g DW) < Z4 (6.75 mg/g DW) < J5 (6.80 mg/g DW) < G6 (7.15 mg/g DW) < T7 (7.83 mg/g DW) < YXM2 (7.85 mg/g DW) < R2 (9.32 mg/g DW) < T6 (12.95 mg/g DW) < B-2–8 (15.76 mg/g DW) < GSW (16.19 mg/g DW) < QM (16.24 mg/g DW) < J4-1 (17.71 mg/g DW) < Y2 (23.13 mg/g DW) < YD (25.35 mg/g DW) < DS (27.16 mg/g DW) < Y1 (38.48 mg/g DW) < CGS (51.28 mg/g DW).

In contrast, the bound phenolic content in the samples followed a quite different order: G8 (0.20 mg/g DW) < T7 (0.24 mg/g DW) < BR60 (0.25 mg/g DW) < 7403 (0.27 mg/g DW) < J5 (0.29 mg/g DW) < S54 (0.31 mg/g DW) < G6 (0.33 mg/g DW) < R2 and G1 (0.34 mg/g DW) < YD (0.37 mg/g DW) < YXM2 (0.39 mg/g DW) < Z1 (0.44 mg/g DW) < QM (0.51 mg/g DW) < B-2–8 (0.51 mg/g DW) < CGS (0.53 mg/g DW) < T6 (0.54 mg/g DW) < Y1 (0.64 mg/g DW) < Y2 (0.71 mg/g DW) < J4-1 (0.75 mg/g DW) < Z4 (0.75 mg/g DW) < DS (0.83 mg/g DW) < Z2 (0.87 mg/g DW) < GSW (1.11 mg/g DW).

Overall, the results revealed that CGS from Taiwan possessed the highest total and free phenolic contents, whereas G1 from North China displayed the lowest values among the 23 cultivars studied. In contrast, the bound phenolic contents of the mulberry cultivars followed a different trend, with the highest and lowest values observed for GSW and G8, respectively. The results further demonstrate that the phenolic compounds present in mulberry leaves predominantly exist in the free state. Furthermore, the total phenolic contents of mulberry leaf cultivars from the same geographical area varied considerably; for instance, GSW, DS, Z4, QM, J4-1, Y2, and Y1 displayed the highest free phenolic contents of the specimens from North China, South China, Southwest China, Thailand, Japan, India, and Vietnam, respectively.

### Free and bound phenolic profiles

**Fig. 2:**
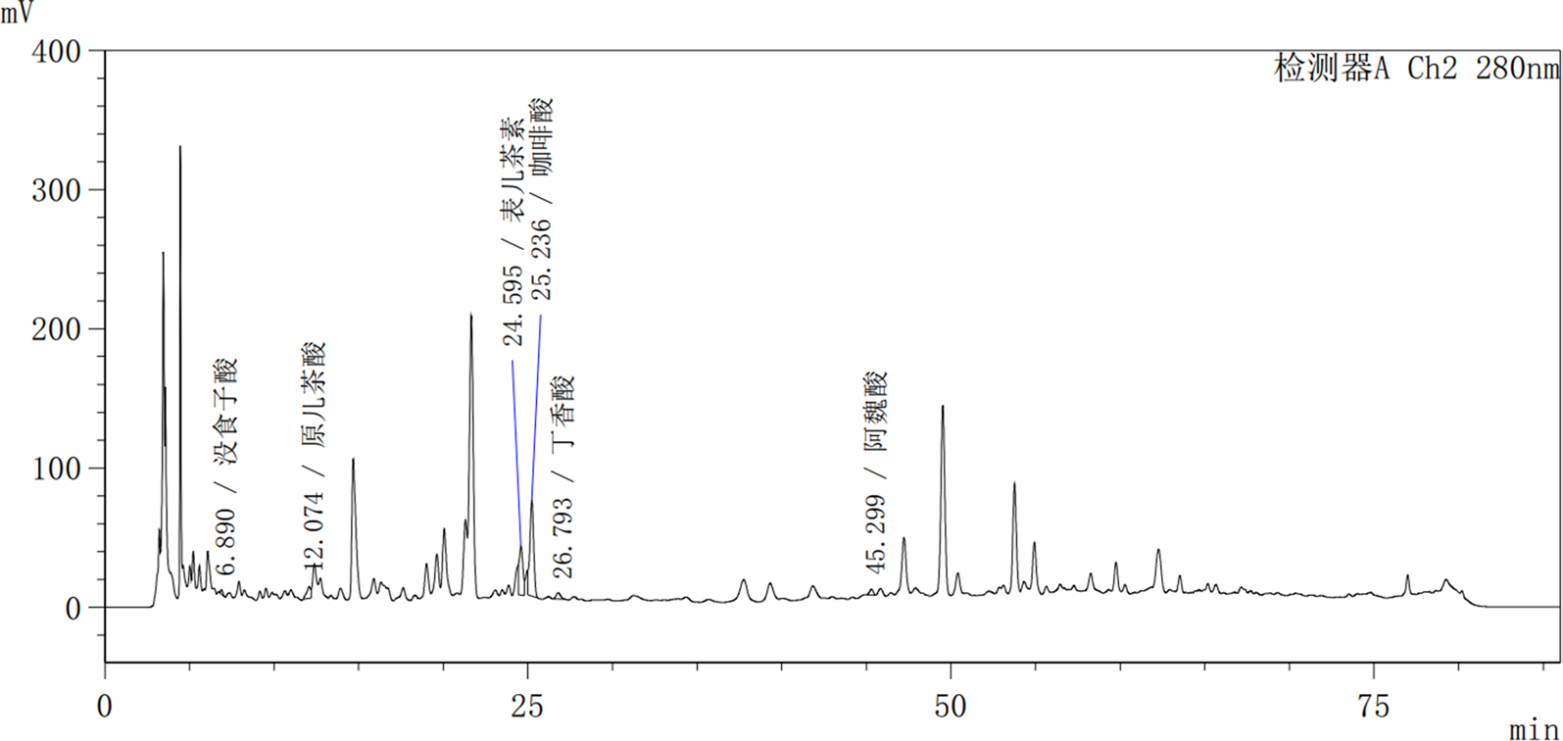
HPLC Chromatograms at 280 nm of the leaves of the mulberry varieties from Z2

**Fig. 3:**
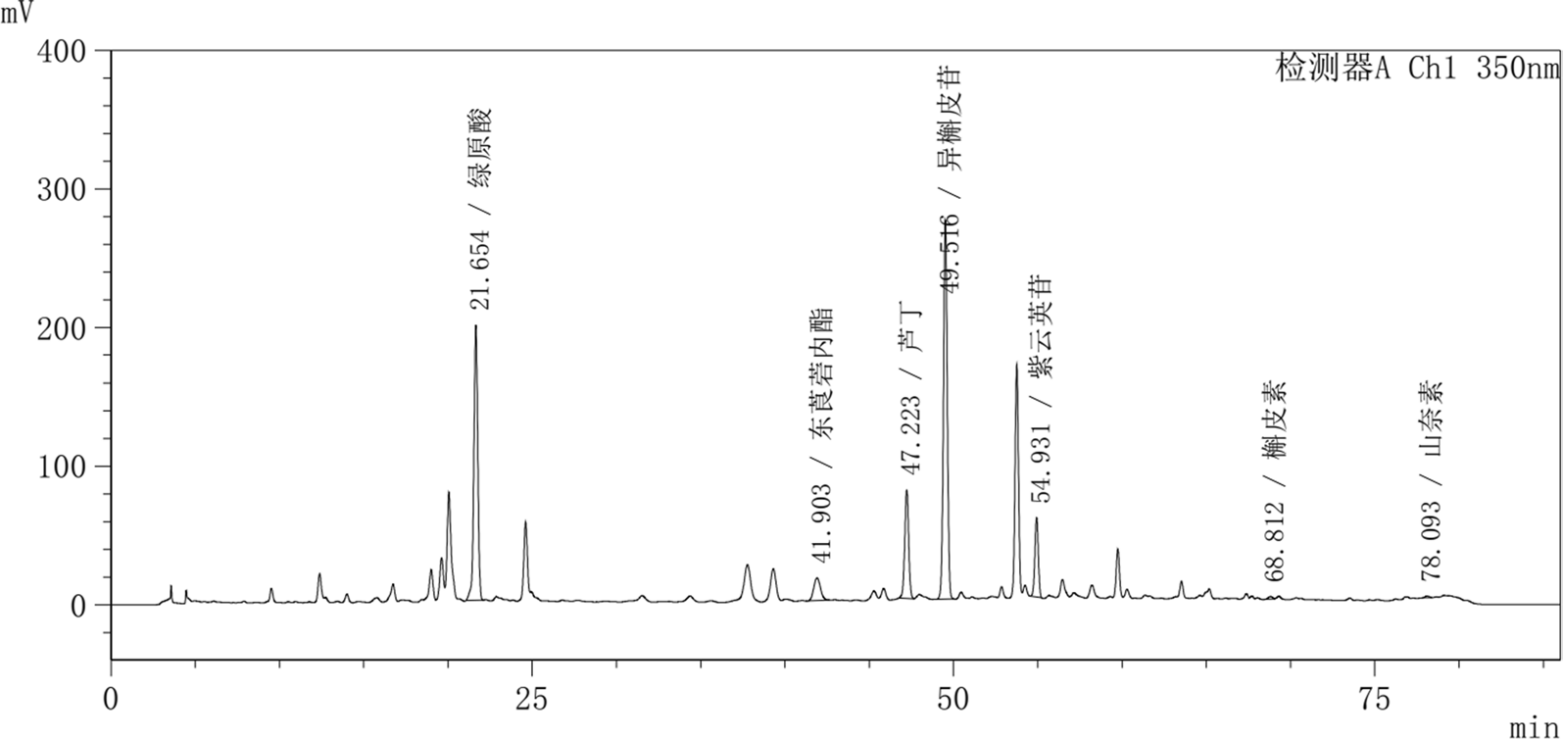
HPLC Chromatograms at 350 nm of the leaves of the mulberry varieties from Z2

Chromatograms of phenolic compounds of the leaves of the mulberry varieties from Z2 are shown in Figs. 2 and 3. Identify the main phenolic compounds by comparing the retention time and other data of the standards. In addition, phenolic compounds are quantified by using a corresponding standard curve with a higher correlation value. Tables 6 and 7 summarize the contents of each phenolic substance in the mulberry leaf samples in the free and bound states, respectively. As shown in Table 6, HPLC analysis revealed the presence of 11 phenolic compounds in the free state, of which ChA, Epi, CaA, Rut, Iso, and Ast together accounted for over 50% of the free phenolic content. This result is consistent with previous reports by Zou et al. [40] and Onogi et al. [25]. The values of ChA ranged from 1.396 to 44.627 mg/g DW and essentially determined the free phenolic content of the mulberry leaves. The leaves of the mulberry varieties from Vietnam (YD and Y1) and Taiwan (CGS) were found to be especially rich in ChA. As mentioned in previous reports [17, 28], ChA can serve as an antioxidant in vitro and may hinder the formation of mutagenic and carcinogenic *N*-nitroso compounds by inhibiting *N*-nitrosation reactions. Furthermore, ChA was reported to inhibit the oxidation of low-density lipoprotein in vitro and could protect against cardiovascular disease [19]. Hence, ChA could potentially be extracted from mulberry leaves and refined for use as a medicine to treat human diseases.

**Table 6:** Free phenolic compounds detected in the 23 mulberry leaf samples by HPLC

Compound (mg/g DW)	ChA	Epi	CaA	Rut	Iso	Ast
G1	1.396 ± 0.128^j^	0.3 ± 0.039^gh^	0.126 ± 0.016^ cd^	0.073 ± 0.01^ k^	0.178 ± 0.017^ k^	0.123 ± 0.008^ lm^
G6	4.268 ± 0.65^ghij^	0.73 ± 0.096^bc^	0.124 ± 0.016^ cd^	0.309 ± 0.043^ijk^	0.854 ± 0.089^f^	0.756 ± 0.071^ef^
G8	2.402 ± 0.242^ij^	0.388 ± 0.052^efgh^	0.145 ± 0.03^c^	0.285 ± 0.055^ijk^	0.513 ± 0.063^fghijk^	0.183 ± 0.042^ijklm^
GSW	12.603 ± 1.26^d^	0.467 ± 0.061^e^	0.047 ± 0.006^fgh^	1.068 ± 0.085^de^	1.494 ± 0.194^de^	0.472 ± 0.058^ g^
R2	21.127 ± 1.93^c^	0.81 ± 0.088^ab^	0.235 ± 0.049^ab^	1.136 ± 0.179^d^	2.093 ± 0.216^b^	1.553 ± 0.228^a^
7403	3.122 ± 0.301^hij^	0.3 ± 0.025^gh^	0.037 ± 0.003^gh^	0.181 ± 0.019^ijk^	0.29 ± 0.028^ijk^	0.274 ± 0.022^hijkl^
DS	7.494 ± 0.614^e^	0.338 ± 0.036^fgh^	0.223 ± 0.031^b^	0.409 ± 0.033^hijk^	0.447 ± 0.041^ghijk^	0.279 ± 0.052^hijkl^
B-2-8	13.766 ± 1.593^d^	0.352 ± 0.043^efgh^	0.045 ± 0.006^gh^	0.252 ± 0.046^ijk^	0.733 ± 0.061^ fg^	0.46 ± 0.039^ g^
Z1	3.49 ± 0.416^ghij^	0.341 ± 0.035^fgh^	0.071 ± 0.009^efg^	0.134 ± 0.025^jk^	0.341 ± 0.045^hijk^	0.067 ± 0.009^ m^
Z2	3.42 ± 0.333^ghij^	0.376 ± 0.071^efgh^	0.118 ± 0.013^ cd^	0.289 ± 0.044^ijk^	0.681 ± 0.071^fgh^	0.14 ± 0.026^klm^
Z4	4.9 ± 0.433^fghi^	0.395 ± 0.052^efg^	0.277 ± 0.053^a^	0.305 ± 0.043^ijk^	0.631 ± 0.056^fghi^	0.16 ± 0.032^jklm^
CGS	44.627 ± 4.149^a^	0.295 ± 0.056^gh^	0.042 ± 0.005^gh^	2.248 ± 0.417^c^	3.187 ± 0.476^a^	0.754 ± 0.1^ef^
T6	8.47 ± 1.062^e^	0.432 ± 0.073^ef^	0.091 ± 0.011^def^	1.263 ± 0.194^d^	1.881 ± 0.35^bc^	0.672 ± 0.073^f^
T7	5.627 ± 0.737^efgh^	0.756 ± 0.07^bc^	0.032 ± 0.004^gh^	0.411 ± 0.045^hijk^	0.582 ± 0.049^fghij^	0.354 ± 0.056^ghi^
QM	2.695 ± 0.326^hij^	0.313 ± 0.029^gh^	0.041 ± 0.003^gh^	0.416 ± 0.032^hij^	0.412 ± 0.021^ghijk^	0.315 ± 0.033^ghijk^
BR60	2.217 ± 0.242^ij^	0.302 ± 0.032^gh^	0.059 ± 0.005^fgh^	0.378 ± 0.042^hijk^	0.382 ± 0.038^ghijk^	0.257 ± 0.025^hijkl^
S54	12.413 ± 1.943^d^	0.601 ± 0.065^d^	0.09 ± 0.009^def^	0.931 ± 0.09^def^	1.231 ± 0.159^e^	0.861 ± 0.114^de^
J4-1	12.969 ± 1.612^d^	0.923 ± 0.098^a^	0.25 ± 0.044^ab^	0.771 ± 0.074^efg^	1.555 ± 0.279^cde^	1.044 ± 0.143^bc^
J5	4.577 ± 0.509^fghi^	0.276 ± 0.041^ h^	0.053 ± 0.009^fgh^	0.666 ± 0.097^fgh^	0.619 ± 0.084^fghi^	0.367 ± 0.056^gh^
Y2	18.341 ± 2.335^c^	0.666 ± 0.092^ cd^	0.109 ± 0.014^cde^	1.165 ± 0.133^d^	1.739 ± 0.165^bcd^	0.934 ± 0.121^ cd^
YXM2	6.429 ± 0.437^efg^	0.282 ± 0.033^gh^	0.133 ± 0.024^ cd^	0.507 ± 0.047^ghi^	0.237 ± 0.029^ k^	0.136 ± 0.022^ lm^
YD	19.797 ± 2.348^c^	0.153 ± 0.026^i^	0.017 ± 0.002^ h^	3.57 ± 0.468^b^	1.332 ± 0.135^e^	0.336 ± 0.062^ghij^
Y1	29.099 ± 2.512^b^	0.367 ± 0.037^efgh^	0.035 ± 0.005^gh^	4.397 ± 0.352^a^	3.077 ± 0.363^a^	1.128 ± 0.184^b^

Compound (mg/g DW)	PrA	Que	GaA	Sco	BeA
G1	0.029 ± 0.005^gh^	ND	0.018 ± 0.002^c^	0.002 ± 0^c^	ND
G6	ND	ND	0.024 ± 0.004^b^	ND	0.03 ± 0.004^a^
G8	0.032 ± 0.005^defgh^	ND	0.017 ± 0.002^ cd^	ND	0.021 ± 0.004^a^
GSW	ND	0.018 ± 0.002^a^	0.007 ± 0.001^ghi^	ND	ND
R2	0.033 ± 0.003^defg^	0.016 ± 0.002^abc^	0.002 ± 0^kl^	0.004 ± 0^c^	ND
7403	0.036 ± 0.003^bcdefg^	0.015 ± 0.002^abc^	0.037 ± 0.004^a^	ND	ND
DS	0.034 ± 0.004^cdefg^	0.013 ± 0.003^bc^	0.011 ± 0.002^ef^	ND	ND
B-2–8	0.03 ± 0.004^gh^	0.014 ± 0.003^bc^	0.004 ± 0.001^hijkl^	ND	ND
Z1	0.032 ± 0.005^defgh^	0.017 ± 0.002^ab^	0.018 ± 0.002^ cd^	ND	ND
Z2	0.044 ± 0.007^bcd^	0.015 ± 0.002^abc^	0.005 ± 0.001^hijk^	0.006 ± 0.001^c^	ND
Z4	0.048 ± 0.009^ab^	0.013 ± 0.002^bc^	0.01 ± 0.001^ fg^	ND	ND
CGS	ND	0.013 ± 0.003^bc^	ND	ND	ND
T6	0.03 ± 0.007^fgh^	0.013 ± 0.003^bc^	0.003 ± 0.001^jkl^	ND	ND
T7	0.031 ± 0.007^efgh^	ND	0.007 ± 0.001^gh^	ND	ND
QM	0.042 ± 0.004^bcdef^	0.012 ± 0.001^c^	0.005 ± 0.001^hij^	0.014 ± 0.002^c^	ND
BR60	0.056 ± 0.007^a^	ND	0.003 ± 0^ijkl^	0.015 ± 0.002^c^	ND
S54	0.043 ± 0.009^bcde^	ND	0.005 ± 0.001^hijk^	ND	ND
J4-1	0.025 ± 0.006^gh^	0.016 ± 0.002^abc^	0.005 ± 0.001^hijk^	0.005 ± 0.001^c^	ND
J5	0.045 ± 0.005^abc^	0.012 ± 0.002^c^	0.006 ± 0.001^hij^	0.07 ± 0.008^b^	ND
Y2	0.02 ± 0.005^ h^	0.015 ± 0.002^abc^	0.001 ± 0^ l^	ND	ND
YXM2	0.029 ± 0.006^gh^	ND	0.014 ± 0.002^de^	ND	ND
YD	ND	ND	ND	ND	ND
Y1	ND	0.014 ± 0.003^abc^	ND	0.13 ± 0.023^a^	ND

*ND* not detected

Different letters indicate significant differences in free phenolic compounds detected in the 23 mulberry leaf samples by HPLC (P < 0.05)

Values with no letters in common in each column are significantly different (P < 0.05), n = 3

**Table 7:** Bound phenolic compounds detected in the 23 mulberry leaf samples by HPLC

Compound (mg/g DW)	CaA	Iso	Ast	PrA	FeA
G1	0.125 ± 0.021^j^	0.086 ± 0.008^fgh^	0.049 ± 0.006^ef^	0.02 ± 0.003^ef^	0.013 ± 0.003^abc^
G6	0.133 ± 0.022^j^	0.08 ± 0.008^fghi^	0.068 ± 0.008^d^	0.016 ± 0.002^efgh^	0.005 ± 0.001^jklm^
G8	0.141 ± 0.024^j^	ND	0.027 ± 0.003^ghij^	ND	0.011 ± 0.002^cdef^
GSW	0.531 ± 0.04^a^	0.412 ± 0.04^a^	0.059 ± 0.008^de^	0.046 ± 0.007^a^	0.012 ± 0.003^abcd^
R2	0.317 ± 0.024^def^	0.25 ± 0.022^c^	0.185 ± 0.023^a^	0.016 ± 0.003^efgh^	0.011 ± 0.002^bcde^
7403	0.123 ± 0.018^j^	0.047 ± 0.005^ij^	0.04 ± 0.006^ fg^	0.018 ± 0.003^defg^	0.014 ± 0.003^ab^
DS	0.201 ± 0.021^hj^	0.044 ± 0.006^ij^	0.017 ± 0.003^ij^	0.013 ± 0.002^gh^	0.013 ± 0.002^abc^
B-2–8	0.263 ± 0.031^ fg^	0.127 ± 0.02^e^	0.072 ± 0.007^d^	0.003 ± 0^j^	0.004 ± 0^klm^
Z1	0.284 ± 0.035^ fg^	0.081 ± 0.011^fghi^	0.017 ± 0.003^ij^	0.023 ± 0.003^ cd^	0.007 ± 0.001^hijkl^
Z2	0.365 ± 0.039^ cd^	0.357 ± 0.035^b^	0.032 ± 0.005^ghi^	0.032 ± 0.004^b^	0.009 ± 0.001^efghi^
Z4	0.359 ± 0.038^cde^	0.255 ± 0.034^c^	0.036 ± 0.004^fgh^	0.028 ± 0.003^bc^	0.01 ± 0.001^defg^
CGS	0.383 ± 0.034^c^	0.075 ± 0.006^ghi^	0.022 ± 0.002^hij^	0.008 ± 0^ij^	0.007 ± 0.001^ghijk^
T6	0.198 ± 0.024^hi^	0.198 ± 0.028^d^	0.063 ± 0.005^de^	0.012 ± 0.002^hi^	0.009 ± 0.001^defghi^
T7	0.094 ± 0.01^j^	0.057 ± 0.007^ghij^	0.033 ± 0.003^ghi^	0.014 ± 0.002^gh^	0.014 ± 0.002^abc^
QM	0.108 ± 0.012^j^	0.046 ± 0.005^ij^	0.018 ± 0.002^ij^	0.016 ± 0.001^efgh^	0.007 ± 0^ijklm^
BR60	0.147 ± 0.017^ij^	0.062 ± 0.007^ghij^	0.022 ± 0.003^hij^	0.018 ± 0.002^defg^	0.004 ± 0^ m^
S54	0.307 ± 0.03^ef^	0.116 ± 0.013^ef^	0.028 ± 0.004^ghij^	0.016 ± 0.002^fgh^	0.008 ± 0^fghi^
J4-1	0.314 ± 0.026^def^	0.209 ± 0.025^d^	0.135 ± 0.019^b^	0.022 ± 0.003^d^	0.015 ± 0.002^a^
J5	0.138 ± 0.01^j^	0.051 ± 0.007^hij^	0.02 ± 0.001^ij^	0.012 ± 0.002^hi^	0.007 ± 0.001^ghij^
Y2	0.307 ± 0.031^ef^	0.22 ± 0.024^ cd^	0.099 ± 0.011^c^	0.021 ± 0.002^de^	0.009 ± 0.001^efghi^
YXM2	0.273 ± 0.022^ fg^	0.032 ± 0.004^j^	0.014 ± 0.002^j^	0.011 ± 0.002^hi^	0.01 ± 0.001^defgh^
YD	0.23 ± 0.02^gh^	0.06 ± 0.006^ghij^	0.016 ± 0.002^j^	0.003 ± 0^j^	0.008 ± 0.001^ghij^
Y1	0.468 ± 0.045^b^	0.09 ± 0.006^efg^	ND	0.006 ± 0.001^j^	0.004 ± 0^ lm^

Compound (mg/g DW)	SyA	Quer	Rut	GaA	BeA
G1	0.004 ± 0.001^ l^	ND	ND	ND	0.002 ± 0^a^
G6	0.006 ± 0.001^jkl^	ND	0.01 ± 0.001^ g^	ND	0.001 ± 0^b^
G8	0.005 ± 0.001^jkl^	ND	ND	0.001 ± 0^e^	0.001 ± 0^b^
GSW	0.01 ± 0.002^ghi^	0.008 ± 0.001^a^	0.032 ± 0.004^a^	0.001 ± 0^ cd^	ND
R2	0.021 ± 0.003^c^	0.007 ± 0.001^ab^	ND	ND	ND
7403	0.003 ± 0^ l^	0.007 ± 0.001^ab^	0.01 ± 0.002^ g^	0.001 ± 0^e^	ND
DS	0.009 ± 0.001^hij^	0.007 ± 0.001^ab^	0.012 ± 0.002^efg^	0.001 ± 0^e^	ND
B-2–8	0.014 ± 0.002^ef^	0.006 ± 0.001^b^	ND	ND	ND
Z1	0.007 ± 0.001^jkl^	0.007 ± 0.001^ab^	0.011 ± 0.002^ fg^	0.003 ± 0^a^	ND
Z2	0.008 ± 0.001^ijk^	0.007 ± 0.001^ab^	0.02 ± 0.002^b^	0.001 ± 0c	ND
Z4	ND	0.007 ± 0.001^ab^	0.017 ± 0.002^bcd^	0.001 ± 0^b^	ND
CGS	0.019 ± 0.002^ cd^	ND	0.017 ± 0.002^bcd^	0.001 ± 0^e^	ND
T6	0.014 ± 0.002^ef^	0.007 ± 0^ab^	0.034 ± 0.004^a^	0.001 ± 0^de^	ND
T7	0.004 ± 0.001^ l^	0.006 ± 0.001^b^	ND	0.001 ± 0^de^	ND
QM	0.005 ± 0^kl^	0.007 ± 0.001^ab^	0.013 ± 0.002^defg^	0.001 ± 0^cde^	ND
BR60	0.007 ± 0.001^ijk^	ND	0.016 ± 0.002^bcde^	0.001 ± 0^e^	ND
S54	0.013 ± 0.002^ fg^	ND	ND	0.001 ± 0^e^	ND
J4-1	0.017 ± 0.001^de^	0.007 ± 0.001^ab^	0.018 ± 0.002^bc^	0.001 ± 0^e^	ND
J5	0.008 ± 0.001^ij^	0.007 ± 0.001^ab^	0.015 ± 0.001^cdef^	ND	ND
Y2	0.027 ± 0.002^b^	ND	ND	0.001 ± 0^cde^	ND
YXM2	0.012 ± 0.001^fgh^	ND	0.016 ± 0.002^bcde^	0.001 ± 0^cde^	ND
YD	0.021 ± 0.002^c^	ND	0.031 ± 0.002^a^	ND	ND
Y1	0.035 ± 0.004^a^	0.007 ± 0.001^ab^	0.033 ± 0.004^a^	ND	ND

*ND* not detected

Different letters indicate significant differences in bound phenolic compounds detected in the 23 mulberry leaf samples by HPLC(P < 0.05)

Values with no letters in common in each column are significantly different (P < 0.05), n = 3

As shown in Table 6, the leaves of the various mulberry cultivars contained different amounts of individual phenolic compounds. However, not all of the 11 phenolic compounds were detected in all samples; for example, BeA was only observed in G6 and G8, while Sco was only found in G1, R2, Z2, QM, BR60, J4-1, J5, Y1. Although none of the 23 cultivars contained all 11 components, J4-1,R2, Z2 and J5 were the richest in free phenolic compounds among the samples tested, with ten components detected. In contrast, YD contained the smallest variety of phenolic compounds, with only six of the components detected.

As shown in Table 7, CaA, Iso, Ast, PrA, FeA, and SyA accounted for the majority of the bound phenolic content for most of the samples, although Iso and PrA were not detected in G8 and SyA was not detected in Z4. Among these six main bound phenolic compounds, CaA was the major component. Previous studies have indicated that CaA, as an α-tocopherol protectant in low-density lipoprotein, is a superior antioxidant compared with FeA, which can also serve as a potent antioxidant to eliminate free radicals and singlet oxygen [15, 24].GSW, 7403, DS, Z1, Z2, T6, QM and J4-1were the richest among the 23 cultivars in terms of bound phenolic compounds.

The data presented in Tables 6 and 7 show that the total phenol contents of Rut (21.472 mg/g DW), Ast (12.700 mg/g DW), ChA (245.249 mg/g DW), and BeA(0.055 mg/g DW) were generally consistent with those determined by Zou et al. [40], who reported a Rut content of 0.1–0.7 mg/g DW, an Ast content of 0.1–0.5 mg/g DW, a ChA content of 0.9–2.1 mg/g DW, and a BeA content of 0–0.2 mg/g DW.

Combined with Tables 6 and 7, only 10 phenolic compounds could be found in the bound phenolic content, which was one phenolic kindless than free phenol. Seven phenolic compounds were detected in both the free and bound fractions, indicating that they occur in mulberry leaves in both forms. Similar to the results for the free phenolic compounds, BeA was detected in the bound fraction for samples G6, G8, and G1. Although the bound phenolic content was relatively low, it cannot be neglected, especially in the case of CaA, because the conjugated forms have been demonstrated to act as more powerful antioxidants in various systems [13, 24].

### Antioxidant activity and its correlation with phenolic content

The antioxidant activities of the free phenolic fractions of the 23 mulberry leaf samples were determined using three separate assays (FRAP, ABTS, and DPPH). As shown in Table 8, the FRAP, ABTS, and DPPH values ranged from 35.13 μmol Fe^2+^/g DW (G1) to 227.8 μmol Fe^2+^/g DW (CGS), from 19.81 μmol TEAC/g DW (7403) to 120.42 μmol TEAC/g DW (Y2), and from 23.11 μmol AEAC/g DW (G1) to 256.63 μmol AEAC/g DW (CGS), respectively. The free phenolic content and antioxidant activity exhibited a certain degree of positive correlation.

**Table 8:** Antioxidant activities of the free phenolic fractions of the 23 mulberry leaf samples

Cultivar	FRAP (μmol Fe^2+^/g DW)	ABTS (μmol TEAC/g DW)	DPPH (μmol AEAC/g DW)
G1	35.13 ± 2.47^ h^	23.53 ± 1.66^gh^	23.11 ± 1.91^i^
G6	70.21 ± 5.63^ef^	32.98 ± 2.64^ g^	55.21 ± 4.43^efg^
G8	47.33 ± 4.95^fgh^	24.97 ± 2.57^gh^	33.87 ± 3.54^gh^i
GSW	108.14 ± 11.13^d^	45.52 ± 4.68^f^	102.77 ± 10.57^d^
R2	66.68 ± 5.95^ef^	30.04 ± 2.68^gh^	40.81 ± 3.64^fghi^
7403	41 ± 3.46^gh^	19.81 ± 1.67^ h^	26.37 ± 2.23^c^
DS	166.13 ± 16.59^c^	81.23 ± 8.11^c^	158.15 ± 15.79^i^
B-2–8	79.97 ± 9.18^ef^	35.74 ± 4.1^ fg^	41.23 ± 4.73^fghi^
Z1	40.72 ± 4.93^gh^	25.08 ± 3.04^gh^	30.04 ± 3.64^hi^
Z2	53.17 ± 5.95^fgh^	28.66 ± 3.21^gh^	42.01 ± 4.7^fghi^
Z4	55.91 ± 5.65^efgh^	32.27 ± 3.26^gh^	44 ± 4.45^fghi^
CGS	227.8 ± 23.25^a^	96.22 ± 9.82^b^	256.63 ± 26.19^a^
T6	129.14 ± 17.87^d^	63.25 ± 8.75^d^	100.27 ± 13.87^d^
T7	63.16 ± 7.85^efg^	28.73 ± 3.57^gh^	43.08 ± 5.36^fghi^
QM	41.26 ± 4.44^gh^	25.09 ± 2.7^gh^	37.15 ± 4^ghi^
BR60	105.83 ± 15.65^d^	48.07 ± 7.11^ef^	97.37 ± 14.4^d^
S54	45.76 ± 4.85^fgh^	24.06 ± 2.55^gh^	28.9 ± 3.07^hi^
J4-1	114.68 ± 14.8^d^	59.61 ± 7.69^de^	66.83 ± 8.62^e^
J5	61.98 ± 5.8^efg^	29.48 ± 2.76^gh^	42.59 ± 3.99^fghi^
Y2	129.24 ± 16.12^d^	120.42 ± 15.02^a^	61.05 ± 7.61^ef^
YXM2	51.81 ± 4.04^fgh^	27.41 ± 2.13^gh^	48.73 ± 3.8^efgh^
YD	196.81 ± 23.75^b^	82.42 ± 9.95^c^	182.94 ± 22.08^b^
Y1	221.99 ± 20.28^a^	94.89 ± 8.67^b^	247.82 ± 22.64^a^

As shown in Table 9, the FRAP values of the bound phenolic fractions of the 23 mulberry leaf samples ranged from 12.74 μmol Fe^2+^/g DW (T7) to 45.93 μmol Fe^2+^/g DW (Y2), which did not vary a lot probably. The ABTS values varied from 5.54 μmol TEAC/g DW (T7) to 18.27 μmol TEAC/g DW (T6), where T6 and T7 originated from the same country (Thailand). The DPPH values ranged from 5.11 μmol AEAC/g DW (T7) to 24.05 μmol AEAC/g DW (Y1).

**Table 9:** Antioxidant activities of the bound phenolic fractions of the 23 mulberry leaf samples

Cultivar	FRAP (μmol Fe^2+^/g DW)	ABTS (μmol TEAC/g DW)	DPPH (μmol AEAC/g DW)
G1	16.5 ± 2.16^ij^	9.34 ± 1.22^fghi^	9.85 ± 1.29^fghi^
G6	14.79 ± 1.98^ij^	6.52 ± 0.88^jk^	7.35 ± 0.99^ijk^
G8	14.52 ± 2.37^ij^	6.56 ± 1.07^jk^	7.23 ± 1.18^ijk^
GSW	32.26 ± 3.09^ cd^	13.37 ± 1.28^ cd^	14.31 ± 1.37^de^
R2	22.71 ± 2.71^fgh^	9.55 ± 1.14^efgh^	8.77 ± 1.05^hij^
7403	13.52 ± 2.04^j^	6.93 ± 1.05^ijk^	7.78 ± 1.18^ijk^
DS	37.2 ± 3.61^bc^	16.01 ± 1.55^ab^	20.89 ± 2.03^b^
B-2-8	39.15 ± 5.06^bc^	15.31 ± 1.98^bc^	17.36 ± 2.24^c^
Z1	17.03 ± 2.19^hij^	8.94 ± 1.15^ghij^	9.73 ± 1.25^ghi^
Z2	23.28 ± 2.55^ fg^	11.81 ± 1.29^def^	11.42 ± 1.25^efgh^
Z4	22.76 ± 2.66^fgh^	11.15 ± 1.3^defg^	9.95 ± 1.16^fghi^
CGS	29.45 ± 2.57^de^	12.38 ± 1.08^d^	15 ± 1.31^ cd^
T6	41.89 ± 5.11^a^b	18.27 ± 2.24^a^	22.52 ± 2.75^ab^
T7	12.74 ± 1.44^j^	5.54 ± 0.63^ k^	5.11 ± 0.58^ k^
QM	16.91 ± 1.92^hij^	8.94 ± 1.01^ghij^	10.02 ± 1.14^fghi^
BR60	26.21 ± 2.76^ef^	11.92 ± 1.26^de^	14.32 ± 1.51^de^
S54	13.65 ± 1.53^j^	6.92 ± 0.77^ijk^	5.99 ± 0.67^jk^
J4-1	28.47 ± 3.04^de^f	13.31 ± 1.42^ cd^	12.72 ± 1.36^def^
J5	17.72 ± 1.71^ghij^	7.75 ± 0.75^hijk^	7.74 ± 0.75^ijk^
Y2	45.93 ± 4.83^a^	14.99 ± 1.58^bc^	20.48 ± 2.15^b^
YXM2	19.64 ± 1.86^ghi^	9.24 ± 0.88^ghi^	11.93 ± 1.13^efg^
YD	26.98 ± 2.45^def^	10.83 ± 0.98^defg^	14.31 ± 1.3^de^
Y1	37.86 ± 3.56^bc^	16.4 ± 1.54^ab^	24.05 ± 2.26^a^

Overall, the results of the three assays revealed a positive correlation between the free and bound phenolic contents and the antioxidant activities. Thus, the correlations between the antioxidant activities and the contents of individual phenolic compounds as determined by HPLC were evaluated to further examine the differences between the 23 cultivars.

### Correlation between antioxidant activities and contents of individual phenolic compounds

Correlation analysis was conducted to determine whether any linear relationships existed between the antioxidant activities, total phenolic content, free phenolic content, bound phenolic content, and content of each phenolic component, and the results are summarized in Tables 10, 11, and 12. Owing to the diversity of the tested cultivars and differences in climate and other factors between different regions, the correlation of the different measured data was also different.

**Table 10:** Correlation coefficients (*R*^2^) for the linear relationships between the total phenolic content, free phenolic content, bound phenolic content and FRAP, DPPH, and ABTS activities

	FRAP^a^	ABTS^b^	DPPH^c^	FRAP^d^	ABTS^e^	DPPH^f^	PC_total_^g^	PC_free_^h^	PC_bound_^i^
FRAP^a^	1	0.8898	0.96	0.6751	0.6437	0.7375	0.795	0.7973	0.1861
ABTS^b^	–	1	0.7676	0.7876	0.6866	0.7878	0.7052	0.7059	0.2342
DPPH^c^	–	–	1	0.5208	0.5338	0.6443	0.7722	0.7755	0.1234
FRAP^d^	–	–	–	1	0.9532	0.9389	0.4926	0.4873	0.4582
ABTS^e^	–	–	–	–	1	0.9495	0.4193	0.4131	0.4749
DPPH^f^	–	–	–	–	–	1	0.4629	0.4606	0.2929
PC_total_^g^	–	–	–	–	–	–	1	0.9998	0.3899
PC_free_^h^	–	–	–	–	–	–	–	1	0.3732
PC_bound_^i^	–	–	–	–	–	–	–	–	1

^a^Ferric reducing antioxidant power of the free phenolic extract

^b^ABTS radical scavenging activity of the free phenolic extract

^c^DPPH radical scavenging activity of the free phenolic extract

^d^Ferric reducing antioxidant power of the bound phenolic extract

^e^ABTS radical scavenging activity of the bound phenolic extract

^f^DPPH radical scavenging activity of the bound phenolic extract

^g^Total phenolic content

^h^Free phenolic content

^i^Bound phenolic content

**Table 11:** Correlation coefficients (*R*^2^) for the linear relationships between the antioxidant activities and contents of each free phenolic component

Compound	ChA	Epi	CaA	Rut	Iso	Ast	PrA	Que	GaA	Sco	FRAP	ABTS	DPPH
ChA	1	0.06109	− 0.15278	0.74647	0.90622	0.62171	− 0.45845	− 0.04528	− 0.54529	0.58749	0.78381	0.69221	0.76781
Epi	–	1	0.43503	− 0.14782	0.23562	0.65714	− 0.41698	0.46368	− 0.09566	− 0.31809	− 0.13601	0.00211	− 0.27078
CaA	–	–	1	− 0.31581	− 0.08295	0.18897	− 0.12246	0.06754	0.05357	− 0.55668	− 0.17605	− 0.07144	− 0.23755
Rut	–	–	–	1	0.77122	0.46117	− 0.25405	− 0.10427	− 0.51705	0.86516	0.81368	0.66942	0.81723
Iso	–	–	–	–	1	0.7687	− 0.34297	0.02399	− 0.57575	0.60393	0.74951	0.67925	0.71518
Ast	–	–	–	–	–	1	− 0.31615	0.15592	− 0.42047	0.23725	0.37703	0.40483	0.27307
PrA	–	–	–	–	–	–	1	− 0.51773	− 0.08846	0.40536	− 0.22612	− 0.39174	0.03339
Que	–	–	–	–	–	–	–	1	0.2282	− 0.41755	− 0.18283	− 0.12509	− 0.22011
GaA	–	–	–	–	–	–	–	–	1	− 0.41773	− 0.52943	− 0.52334	− 0.4482
Sco	–	–	–	–	–	–	–	–	–	1	0.76458	0.7181	0.83016
FRAP	–	–	–	–	–	–	–	–	–	–	1	0.88975	0.96
ABTS	–	–	–	–	–	–	–	–	–	–	–	1	0.76762
DPPH	–	–	–	–	–	–	–	–	–	–	–	–	1

**Table 12:** Correlation coefficients (*R*^2^) for the linear relationships between the antioxidant activities and contents of each bound phenolic component

Compound	CaA	Iso	Ast	PrA	FeA	SyA	Que	Rut	GaA	BeA	FRAP	ABTS	DPPH
CaA	1	0.65768	0.25927	0.37507	− 0.09959	0.62789	0.55234	0.54925	0.22599	− 0.98429	0.46961	0.49066	0.40303
Iso	–	1	0.47181	0.74687	0.22374	0.18262	0.73828	0.43122	0.06553	1	0.32322	0.3395	0.12059
Ast	–	–	1	0.12189	0.25964	0.50799	0.46127	0.17347	− 0.20314	0.34391	0.2905	0.22378	0.09693
PrA	–	–	–	1	0.37708	− 0.33017	0.70274	− 0.01405	0.29171	1	− 0.09967	− 0.046	− 0.21309
FeA	–	–	–	–	1	− 0.3119	0.32327	− 0.07737	− 0.24806	0.47515	− 0.20614	− 0.17771	− 0.27261
SyA	–	–	–	–	–	1	0.09162	0.69018	− 0.17523	− 0.77855	0.6659	0.57881	0.64429
Que	–	–	–	–	–	–	1	0.38495	− 0.04223	–	0.16827	0.21116	0.05313
Rut	–	–	–	–	–	–	–	1	− 0.23894	–	0.72964	0.6797	0.67301
GaA	–	–	–	–	–	–	–	–	1	–	− 0.18081	− 0.11587	− 0.16261
BeA	–	–	–	–	–	–	–	–	–	1	0.98417	0.94582	0.963
FRAP	–	–	–	–	–	–	–	–	–	–	1	0.95325	0.93894
ABTS	–	–	–	–	–	–	–	–	–	–	–	1	0.94952
DPPH	–	–	–	–	–	–	–	–	–	–	–	–	1

As shown in Table 10, between the phenolic contents and the antioxidant activities of the various extracts, the strongest correlation was observed between the free phenolic content and the FRAP values of the free phenolic extracts (*R*^2^ = 0.7978), followed by the relationship between the total phenolic content and the FRAP values of the free phenolic extracts (*R*^2^ = 0.795). The weakest correlation occurred between the bound phenolic content and the DPPH values of the free phenolic extracts (*R*^2^ = 0.1234). Previous studies have indicated that phenolic compounds greatly contribute to the antioxidant activity of mulberry leaves, especially the free phenolic content, which has the greatest influence. A strong correlation was also observed between the bound phenolic content about Rut and the ABTS values of the bound phenolic extracts(R2 = 0.6797), indicating that certain phenolic compounds in the bound phenolic fraction may influence the removal of ABTS free radicals, such as CaA, Iso, and Que (Table 12). However, the correlation between total phenolic content and ABTS, free phenolic content and ABTS were the weakest, indicating that there may have been other substances present in the mulberry leaves that also affected the removal of ABTS free radicals, such as PrA (Table 11).

As shown in Tables 11 and 12, the contents of four phenolic compounds, namely, Iso, Rut, Sco, and ChA, exhibited positive correlations with the antioxidant activities of the free phenolic extracts as determined using all three assays, whereas the other seven phenolic compounds displayed no obvious correlation with the antioxidant activities. The strength of these positive correlations followed the order Rut > ChA > Sco > Iso for the FRAP activity, Sco > Rut > ChA > Iso for the DPPH activity, and Sco > ChA > Iso > Rut for the ABTS activity. However, as Sco was not detected in most of the samples, the free phenolic substances predominantly responsible for the antioxidant activities of the mulberry leaf samples appear to be Iso, ChA, and Rut. The correlation coefficient between the FRAP and DPPH assays was similar in Table 11, which indicates that these two assays were more suitable than the ABTS assay for measuring the antioxidant activity of free phenolic compounds. This may explain why the FRAP and DPPH assays have been more frequently applied to determine the phenolic antioxidant activity in previous studies [32, 39]. With respect to the bound phenolic compounds, SyA and Rut (Rut > SyA) displayed a significant positive correlation with the antioxidant activities of the bound phenolic extracts as determined using all three assays, whereas no obvious correlation was observed for the other compounds.

Overall, the correlation between the phenolic contents and antioxidant activities fluctuated greatly, reflecting that phenolic compounds are the main antioxidants in mulberry leaves.

## Conclusions

The phenolic compositions and antioxidant activities of leaves from 23 mulberry varieties cultivated in several countries and regions were characterized using LC-ESI-QTOF and three separate antioxidant assays (FRAP, ABTS, and DPPH). The results revealed significant differences in the phytochemical contents and antioxidant activities between the samples. The phenolic compounds in mulberry leaves predominantly existed in the free form. CGS from Taiwan displayed the highest phenolic content as well as superior antioxidant activity compared with the other 22 cultivars, as determined by the FRAP, ABTS, and DPPH assays. Furthermore, the obtained results demonstrated that ChA and CaA were the main phenolic compounds in the free and bound fractions, respectively. Iso, ChA, and Rut accounted for the majority of the antioxidant activity in the free phenolic fractions, while SyA and Rut were positively correlated with the antioxidant activity of the bound phenolic fractions. Taken together, these results indicate that the antioxidant activity of mulberry leaves is related to the content and types of phenolic compounds present.

## Data Availability

The datasets generated during and/or analysed during the current study are available from the corresponding author on reasonable request.

## Abbreviations

Rut: Rutin
Que: Quercetin
Quer: Quercitrin
Iso: Isoquercitrin
ChA: Chlorogenic acid
SyA: Syringic acid
FeA: Ferulic acid
CaA: Caffeic acid
Res: Resveratrol
Epi: Epicatechin
Ast: Astragalin
Sco: Scopoletin
Gal: Galangal
CatA: Catechuic acid
VaA: Vanillic acid
BeA: Benzoic acid
GaA: Gallic acid
PrA: Protocatechuic acid
TPTZ: 2,4,6-Tris(2-pyridyl)-S-triazine
DPPH: 1,1-Diphenyl-2-picrylhydrazyl
ABTS: 2,2ʹ-Azino-bis(3-ethylbenzothiazoline-6-sulfonic acid) diammonium salt
trolox: 6-Hydroxy-2,5,7,8-tetramethylchroman-2-carboxylic acid
FRAP: Ferric reducing antioxidant power
AEAC: Ascorbic acid equivalent antioxidant capacity
TEAC: Trolox equivalent antioxidant capacity
RT: Retention time
